# Prevalence of Malocclusion Traits in Primary Dentition, 2010–2024: A Systematic Review

**DOI:** 10.3390/healthcare12131321

**Published:** 2024-07-02

**Authors:** Hanyi Chen, Lude Lin, Jieyi Chen, Fang Huang

**Affiliations:** 1 Hospital of Stomatology, Sun Yat-sen University, Guangzhou 510055, China; chenhy355@mail2.sysu.edu.cn (H.C.); linlt23@mail2.sysu.edu.cn (L.L.); 2 Guangdong Provincial Key Laboratory of Stomatology, Guangzhou 510080, China; 3 Guanghua School of Stomatology, Sun Yat-sen University, Guangzhou 510080, China

**Keywords:** malocclusion, epidemiology, oral health, primary dentition, child

## Abstract

The present review was aimed to describe the prevalence and the regional distribution of malocclusion among preschool children worldwide. Two independent reviewers performed a systematic literature search to identify English publications from January 2010 to May 2024 using PubMed, ISI Web of Science and Embase. Search MeSH key words were “malocclusion”, “primary dentition” and “child, preschool”. The reporting quality was assessed by the modified Newcastle–Ottawa Quality Assessment Scale. We identified 2599 publications and recruited 47 articles. Fourteen of the included studies were conducted in Asia, four in Europe, twenty-eight in South America and one in Africa. The prevalence of malocclusion ranged from 28.4% to 83.9%, and half of the reported prevalences were higher than 50%. The highest percentage was in Asia (61.81%), followed by Europe (61.50%), South America (52.69%) and Africa (32.50%). Statistically significant differences existed in deep overbite, anterior open bite, posterior crossbite, edge-to-edge incisor relationship and distal step between continents (*p* < 0.05). Europe showed the highest prevalence (33.08%) of deep overbite. Africa showed the highest prevalence (18.60%) of anterior open bite. Europe showed the highest prevalence (15.38%) of posterior crossbite. The most common malocclusion traits were increased overjet and deep overbite. To conclude, malocclusion remained prevalent in the primary dentition and varied between countries.

## 1. Introduction

Malocclusion is an abnormality of the teeth or a malrelation between the dental arches. It represents a developmental irregularity of the craniofacial complex and is thus defined as a “handicapping dentofacial anomaly” by the World Health Organization [1]. Malocclusion is considered one of the three major oral diseases that affects the jaws, tongue and facial muscles, compromising dentofacial function as well as facial beauty [2,3,4,5]. Furthermore, it may have negative effects on children’s emotional and social well-being [6,7].

The etiology of malocclusion is multifactorial, including hereditary factors and environmental factors. Dental diseases such as dental caries, pulpal and periapical lesions and dental trauma significantly contribute to malocclusion [1]. Genetic factors have been found to be statistically related to skeletal deformities and malocclusion. Ashwin et al. reported that the growth hormone receptor gene influences the growth of the maxilla and mandible in the sagittal and vertical dimensions [8]. Additionally, genes such as MYO1H, COL2A1, GHR, ARHGAP21 and SNAI3 were found to be associated with skeletal class III malocclusion [9]. Environmental factors, particularly deleterious oral habits, also play a crucial role in the development of malocclusion. Oral habits such as digit sucking, tongue thrusting, sucking or biting of the lips or cheeks, sleep-disordered breathing and unilateral mastication habits could make children more susceptible to malocclusion [10,11,12]. Lin et al. reported that children with a lip-biting habit had a higher prevalence of deep overbite [13]. Moreover, diet and chewing patterns are also related to malocclusion. Boyd et al. summarized that a soft diet could decrease the functionality and growth of the masticatory apparatus, potentially influencing the formation of the dental arch [14]. Nowadays, mouth breathing and its influence on malocclusion are raising concern. Grippaudo et al. reported that mouth breathing was associated with increased overjet, reduced overjet, anterior or posterior crossbite, open bite and the displacement of contact points [15]. These factors may lead to orofacial myofunctional disorder and contribute to malocclusion as underlying pathologies [16]. Other dental diseases, such as cleft lip and palate, can cause significant growth disturbance of the maxilla, leading to skeletal class III malocclusion and cross bite [17]. A systematic review indicated that lower Dental Aesthetic Index value for malocclusion were significantly related to lower mean DMFT scores [18]. Some of these dental diseases have a high prevalence among preschool children, and their impact on the development of malocclusion should be given attention.

Children who have malocclusion in their primary dentition were suggested to be at a higher risk of developing malocclusion in their mixed and permanent dentition. Additionally, those with posterior crossbite in the primary dentition were more likely to develop a posterior crossbite in their early mixed dentition [19]. Therefore, the early diagnosis of deciduous dentition malocclusion may not only aid in preventive or interceptive orthodontics, utilizing children’s growth potential to alleviate symptoms and functional limitations, but also contribute to better occlusion development in the mixed and permanent dentition.

Previous studies reported variability in the prevalence of malocclusion in primary dentition among preschool children. In Brazil (South America), the prevalence was 63.2%, and in southwest Germany (Europe), it was 61.5%, while in mainland China (Asia), it was 51.8% [20,21,22]. A literature review on the prevalence of Angle’s class III malocclusion concluded that differences exist among ethnicities. Asians had the highest prevalence rate, followed by black Nigerians and European-American populations [23]. Considering that malocclusion is a polygenic trait significantly affected by ethnicity, ethnic characteristics should not be ignored in a review on the prevalence of malocclusion [24].

Although some studies have been conducted on the prevalence of malocclusion, a research gap still exists in this area. Comprehensive information regarding the prevalence and the characteristic features of malocclusion in primary dentition around the world is limited. Most studies were conducted in certain regions, resulting in a lack of data in other parts of the world. Understanding the prevalence of malocclusion in primary dentition worldwide may raise the awareness of healthcare professionals and researchers about this oral condition. It may also help public health planners or healthcare professionals to develop appropriate strategies to prevent and manage this oral problem and encourage researchers to focus on identifying the causes and risk factors associated with malocclusion. Therefore, the aim of the present systematic review is to describe the prevalence and the regional distribution of malocclusion in primary dentition among preschool children globally.

## 2. Materials and Methods

This study was registered in PROSPERO and reported following the Preferred Reporting Items for Systematic reviews and Meta-Analyses (PRISMA) guidelines (Supplementary File S1).

### 2.1. Search Strategy

Three electronic databases (PubMed, ISI Web of Science, Embase) were selected to search for peer-reviewed articles published in English from January 2010 to May 2024. Medical Subject Headings (MeSH) terms were used to search for the keywords “malocclusion” (MeSH), “primary dentition” (MeSH) AND “child, preschool” (MeSH). Duplicate records and papers written in languages other than English were excluded.

### 2.2. Study Selection

Two reviewers (HC and LL) independently screened the titles, abstracts and full texts to select eligible studies. Twenty articles were randomly selected to evaluate the intra- and inter-examiner validity. Both reviewers demonstrated high intra-examiner validity with kappa values over 0.9, and the inter-examiner validity statistic was also deemed acceptable (kappa value  =  0.8). Disputes were resolved by involving the third reviewer (JC) for opinions. A group meeting was held, involving all authors, to discuss the indeterminable articles.

Ineligible publications were excluded according to the following criteria:

(1) Study design: review, meta-analysis, longitudinal studies, case–control studies, cohort studies, clinical trials, case report studies, laboratory studies, abstracts, conference proceedings or commentaries;

(2) Participants: studies in which the participants were limited to a particular occupation, population, community group or location;

(3) Outcomes: studies that did not report the prevalence of malocclusion in primary dentition among preschoolers (3 to 5 years old);

(4) Data: studies without original data;

(5) Studies that did not report the most detailed data for which other articles based on the same sample were available.

### 2.3. Data Extraction

The following information was extracted and summarized during the full-text assessment:

(1) Publication details: including first author and publication year;

(2) Research design: including the study site and total sample size;

(3) Details of the target indicators: including diagnosis criteria, occlusal features and malocclusion traits. The diagnostic criteria for occlusal features and malocclusion traits in this review were summarized as follows:

Sagittal relationship of second primary molars

Flush terminal plane: distal surfaces of the upper and lower second primary molars were in the same vertical plane

Distal step: distal surface of the lower second primary molar lay distal to that of the upper second primary molar.

Mesial step: distal surface of the lower second primary molar lay mesial to that of the upper second primary molar.

Sagittal relationship of primary canines

Class I: the distal surface of the lower primary canine and cusp tip of the upper primary canine were in the same vertical plane.

Class II: the distal surface of the lower primary canine lay distal to the cusp tip of the upper primary canine.

Class III: the distal surface of the lower primary canine lay mesial to the cusp tip of the upper primary canine.

Sagittal Anomalies

Overjet: the distance between the incisal edge of the most protruded upper primary incisor and labial surface of the corresponding lower primary incisor. There were no standardized diagnostic criteria for increased overjet. The cutoff points used in different studies included >2 mm, >2.5 mm, >3 mm, >4 mm, >5 mm and >6 mm.

Edge-to-edge incisor relationship: upper and lower incisal edges met edge-to-edge.

Anterior crossbite: upper primary incisor or canine was positioned inside the lingual surfaces of the lower front teeth.

Vertical Anomalies

Deep overbite: lower incisors were covered by the most protruded upper primary incisor. There were no standardized diagnostic criteria for deep overbite (increased overbite), and the reported cutoff points included >2 mm, >3 mm and >4 mm or greater than 40% and 50% of the lower incisors.

Anterior open bite: no vertical overlap was found between the upper and lower primary incisors when the posterior teeth were in contact.

Transversal Anomalies

Posterior crossbite: any lower primary posterior tooth was placed buccal to the upper primary molars.

Scissor bite: upper molars were occluded buccally to the buccal surfaces of the lower molars.

Midline deviation: midline of the mandibular primary incisors showed a deviation from that of the maxillary primary incisors. There were no standardized diagnostic criteria for midline deviation, and the cutoff points included >1 mm and >2 mm.

Space Discrepancies

Crowding: generally diagnosed if overlapping of erupted primary teeth was >2 mm.

### 2.4. Quality Assessment

To assess the publication quality of the selected studies, we adopted the “Newcastle–Ottawa Scale adapted for cross-sectional studies” (Supplementary File S2). Six items, including representativeness, sample size, non-respondents, ascertainment of risk factor (diagnosis), outcome assessment and statistics, were scored between 0 and 8. The quality of the studies was divided into the following three levels: poor (0–2), moderate (3–5) and good (6–8). Prior to the commencement of assessment, the reviewers learned the evaluation criteria and underwent calibration. The assessment was then performed independently by the same reviewers (HC and LL), who demonstrated high inter- and intra-examiner agreements (kappa value  = 0.9 and 0.7, respectively). Disputes were discussed by the two reviewers to reach an agreement.

### 2.5. Data Analysis

Statistical Package for Social Science version 26.0 (SPSS Inc., Chicago, IL, USA) was used for data analysis. Data were summarized as means and standard deviations (SDs). The Kruskal–Wallis test was used to assess the statistical significance of the differences between four continents. Whenever the Kruskal–Wallis test was significant, the Mann-Whitney U test was used for pair-wise comparisons between groups. The level of statistical significance for all tests was set at *p* < 0.05.

## 3. Results

**Figure 1:**
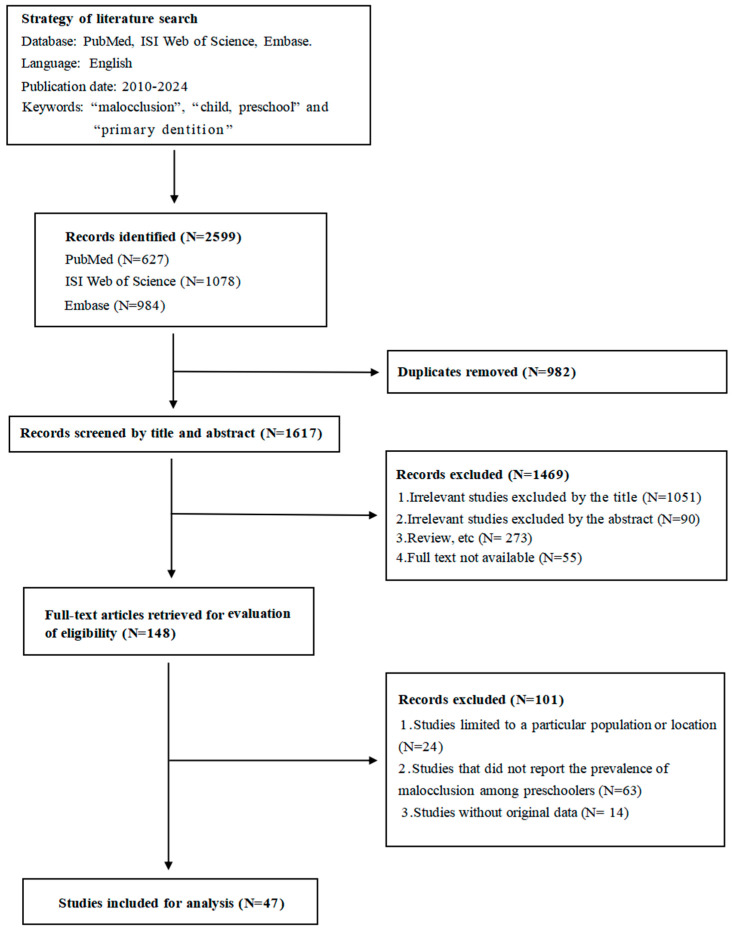
Flowchart of literature search and selection.

We identified and screened 2599 articles (1078 from ISI Web of Science, 627 from PubMed and 984 from Embase) based on the titles and abstracts (Figure 1). The preliminary screening of the titles and abstracts showed that 982 articles were duplicates and 1469 articles failed to meet the inclusion criteria. Full texts of the remaining 148 articles were retrieved for evaluation. Finally, 47 studies met the inclusion criteria and were included in our study. No additional publications were identified from the bibliography of these 148 articles.

The included publications described the occlusal features and malocclusion traits of primary dentition among preschool children in twelve countries/districts from four continents (Table 1 and Table 2). Most of the studies were conducted in South America (N = 28, Brazil, Peru), as well as fourteen in Asia (China, India, Georgia, Japan, Saudi Arabia), four in Europe (Estonia, Germany, Greece, Albania) and one in Africa (Tanzania). Oceania had no publication on this topic during 2010–2024.

The pooled prevalence of malocclusion in primary dentition worldwide was found to be 54.83% (Table 3), with more than half of the studies reporting a prevalence rate of over 50% (Table 1). The most common types of malocclusion were increased overjet (25.18%) and deep overbite (23.79%). Scissor bite (0.30%), edge-to-edge incisor relationship (5.97%) and anterior crossbite (5.73%) were found to be the least prevalent types of malocclusion.

Regarding the geographical distribution of malocclusion prevalence among continents, the highest percentage was in Asia (61.81%), followed by Europe (61.50%), South America (52.69%) and Africa (32.50%). Statistically significant differences were found in the prevalence of deep overbite, anterior open bite, posterior crossbite and edge-to-edge incisor relationship among continents (Table 3, *p* < 0.05). Africa showed the highest prevalence (18.60%) of anterior open bite, and Europe showed the lowest prevalence (4.46%). Statistically significant differences were found between South America and Asia (*p* < 0.001) and between South America and Europe (*p* = 0.002). Conversely, Europe showed the highest prevalence of deep overbite (33.08%), while Africa showed the lowest prevalence (6.30%). Statistically significant differences were found between South America and Europe (*p* = 0.011). Similarly, Europe and South America showed the highest prevalence of posterior crossbite (15.38% and 13.52%, respectively), while Africa showed the lowest prevalence (1.20%). Statistically significant differences were found between South America and Asia (*p* = 0.001) and between Europe and Asia (*p* = 0.007). South America showed the highest prevalence (11.00%) of edge-to-edge incisor relationship and Asia showed the lowest prevalence (2.61%). Statistically significant differences were found between South America and Asia (*p* = 0.021). No statistically significant differences were found in prevalence of increased overjet, anterior crossbite, scissor bite, midline deviation or crowding among the four continents.

The most common terminal plane relationship of the second primary molars was found to be the flush terminal plane (52.33%), followed by the mesial step (32.16%), and the least common was the distal step (17.56%) (Table 3). Almost all articles followed this order except for one study in Estonia with the highest prevalence of mesial step, followed by the flush terminal plane and one study in Belém, Pará, Brazil with the highest prevalence of distal step, followed by the flush terminal plane (Table 2). A study conducted in Brazil, South America, reported the highest prevalence of distal relationship (67.50%), while a study conducted in Peru, South America, reported the highest prevalence of mesial relationship (83.60%). Statistically significant differences were found in the prevalence of distal step relationship between Asia and Europe (*p* = 0.017). The prevalence (29.27%) of distal step relationship in Europe was higher than the prevalence (7.80%) in Asia. No statistically significant differences were found in the prevalence of flush terminal plane and mesial relationship among the four continents.

The most common type of canine relationship was found to be class I (72.49%), followed by class II (17.30%), and the least common was class III (6.60%) (Table 3), except for one study conducted in Hong Kong, which reported class III as the second most common (Table 2). Europe was reported to have the highest prevalence of class II (21.71%), while South America showed the highest prevalence of class III (7.83%). No statistically significant differences were found in the prevalence of canine relationship among the four continents.

The results of the quality assessment of the included studies are presented in Table 4. The total quality score ranged from 2 to 8. Thirty-three studies (70%) were considered of good quality, thirteen (28%) of moderate quality and only one (2%) of poor quality.

## 4. Discussion

This study reported the prevalence and the regional distribution of malocclusion among preschool children worldwide. The pooled prevalence of malocclusion in primary dentition among preschool children worldwide was found to be as high as 54%. Furthermore, the results indicated a high level of variation in the prevalence of deciduous dentition malocclusion across different countries, which may be attributed to differences in socio-environmental factors, ethnic groups, diagnostic criteria and study design.

Our study found statistically significant differences in deep overbite (increased overbite), anterior open bite, posterior crossbite and edge-to-edge incisor relationship between continents. Deep overbite was the second most prevalent type of malocclusion in primary dentition regarding the regional distribution, with the highest prevalence of anterior open bite reported in Africa (18.6%) and the lowest in Europe (4.46%). Furthermore, Africa was the region with the lowest prevalence of deep overbite (6.30%). A previous study suggested that epigenetic regulation determined the skeletal muscle fiber phenotypes and bone growth, which could affect the development of malocclusion [68]. Küchler et al. found that the expression levels of the HDAC4 gene showed a 7-fold increase and that those of the KAT6B gene showed a 2.6-fold increase in subjects with a deep overbite when compared with those with an anterior open bite [69]. Another study revealed that the “C” allele for the C^−1306^T polymorphism and the “T” allele for the C^−1562^T polymorphism in the promoter of the MMP-2 gene were more common in blacks than in whites and suggested that genes play an important role in the etiology of open bite [70]. In addition, another study revealed that East Africans have both skeletal and neuromuscular predispositions to anterior open bite based on skeletal patterns and swallowing action, making people in East Africa more susceptible to treatment-related open bite [71]. Apart from hereditary factors, children with pacifier-sucking habits and lip-biting habits were reported to be more susceptible to open bite and overbite [72].

Regarding posterior crossbite, it was found to be the most prevalent in Europe (15.38%) and South America (13.52%) and the least prevalent in Africa (1.20%). This could be explained by the fact that the Caucasian population generally has a higher prevalence of posterior crossbite than the African and Asian populations [73]. Further analysis showed that the Brazilian population had a significantly greater incidence rate of anterior open bite and posterior crossbite. Brazilians are characterized by a considerable degree of racial miscegenation, including Amerindians, Brazilian African descendants and Latin/European Caucasians [44,52,53]. Therefore, the results may be explained by the diversity of ethnic groups among the Brazilian population. However, there were also studies suggesting that differences in habits were responsible for differences in the prevalence of posterior crossbite between different ethnic groups [74,75]. Non-nutritive sucking habits such as the long-term use of pacifiers or finger sucking and mouth breathing may result in posterior crossbite [76]. Conversely, children who were breastfed for six months or longer had a lower incidence of posterior crossbite in both deciduous and mixed teeth [77]. Moreover, long-term unilateral mastication caused by deep caries may also lead to posterior crossbite [1]. It was reported that the prevalence of early childhood caries in South America ranked second among continents, while Africa had the lowest prevalence [78], which is consistent with the prevalence of posterior crossbite. Further studies are needed to characterize the ethnic and regional distribution of malocclusion in primary dentition.

Anterior crossbite was found to be one of the least prevalent types of malocclusion in this study. However, with respect to regional distribution, Asia had the highest prevalence (7.35%). This might be related to genetically inherited characteristics of anterior crossbite [79]. For the Chinese population, some studies revealed that two single-nucleotide polymorphisms (rs2738, rs229038) of *ADAMTS1* were associated with mandibular prognathism (MP) [80]. *TGFB3*, *LTBP2* [81], *EPB41* [82], *EVC* and *EVC2* [83] are considered candidate functional genes that could confer susceptibility to MP. Additionally, rs13317 in *FGFR1*, rs149242678 in *FGF20* and rs79176051 *FGF12* in the (FGF/FGFR) signaling pathway also showed significant associations with MP [84]. In other Asian populations, the *Matrilin-1* polymorphism haplotype TGC in the Korean population [85] and a rare, non-synonymous SNV of *BEST3* in the Japanese population had a risk effect on MP. However, the *Gly1121Ser* variant in the *ARHGAP21* gene was found to be shared by most MP individuals included in a study, and it is rare in the Caucasian population [86].

Increased overjet and deep overbite are the two most prevalent types of malocclusion in primary dentition (25.18% and 23.79%, respectively). However, it is worth nothing that the cutoff point used for the diagnosis of increased overjet and deep overbite was divergent among the included studies. Increased overjet and deep overbite could be defined as >2 mm [41,42,45], >3 mm [26,30,32] and >4 mm, respectively [37]. Therefore, caution should be taken when comparing the data of one study with another.

This study also found that Europe had a significantly higher prevalence of distal step compared to Asia. A longitudinal study performed by Bishara observed that distal step in the deciduous dentition tend to develop into a class II molar relationship in the permanent dentition [87]. A systematic review by Alhammadi mentioned that the Caucasian population showed the highest prevalence of class II in permanent dentition, which may partly explain the result [79]. South America showed a significantly higher prevalence of edge-to-edge incisor relationship than Asia in the present study. No relevant reports were found, and more studies are required to identify the patterns of edge-to-edge incisor relationship prevalence by different regions and races.

The present study has some inherent limitations. First, the number of included articles in certain continents was insufficient, and thus, using data from a specific city or region to represent the situation in the continent may not be precise. Second, the cutoff point used for increased overjet and deep overbite was divergent among the included articles, which may lead to inaccurate estimates of prevalence and deviated analysis. Moreover, the results of different studies cannot be compared or combined to provide a more comprehensive understanding of this oral condition. So far, there is no standardized comprehensive index to assess malocclusion in primary dentition [36]. Therefore, there is an urgent need to establish standardized diagnostic criteria to promote consistency and accuracy in diagnosing malocclusion in primary dentition.

This systematic review has multiple strengths, such as the adoption of three main databases, namely PubMed, ISI Web of Science and Embase, for searching for publications. PubMed is a database maintained by the National Center for Biotechnology Information at the U.S. National Library of Medicine and contains more than 35 million citations and abstracts of biomedical literature [88]. By compensating for the diversity in medical terminology, MeSH terms and subheadings were adopted to make PubMed searches more sensitive and minimize false-negative (missed) hits. Antoher database used in this study for literature search was the ISI Web of Science. It contains more than 171 million records, including more than 34,000 journals indexed [89]. Embase (Excerpta Medica Database) is a biomedical and pharmacological database produced by Elsevier B.V. and containing more than 41 million records, including articles from more than 8100 journals published worldwide [90]. The literature search could cover a wide range of citation-indexed journals by using these three databases in this study. These journals are generally believed to publish high-quality research. Moreover, only epidemiological surveys were included in this study to generalize the results. We excluded cohort and randomized clinical studies because these studies mostly recruited children from specific groups. In addition, this study adopted several strategies to reduce bias in the quality assessment of the selected studies, including adopting standardized evaluation criteria (the NOS scale), calibration before the commencement of assessment, assessing independently by two raters who maintained a high level of validity and continuing to review the study and feedback by holding group meetings. In summary, the findings of the present study could be a reminder to both dental educators and policymakers that the prevalence rates of malocclusion remain high among preschool children worldwide. Regarding the variations in the prevalence of different types of malocclusion among continents, national oral health policies should be developed according to their respective characteristics.

## 5. Conclusions

Based on the 47 included studies published in the past 15 years (2010–2024), malocclusion in primary dentition is still prevalent worldwide and varies across countries and regions, highlighting the necessity of proactive interventions. There is a statistically significant difference in the prevalence of deep overbite, anterior open bite, posterior crossbite, edge-to-edge incisor relationship and distal step between continents. Africa showed the highest prevalence of anterior open bite, while Europe had the highest prevalence of posterior crossbite. The Brazilian population had a significantly greater incidence rate of anterior open bite and posterior crossbite compared to other continents.

## Figures and Tables

**Table 1:** Summary of sagittal anomalies, vertical anomalies, transversal anomalies and space discrepancies in the included studies.

Region, Authors	Year	Study Site	Total Sample Size	Mal- Occlusion (%)	Sagittal Anomalies			Vertical Anomalies		Transversal Anomalies				Space Discrepancies
					Increased Overjet	Edge-to- Edge Incisor Relationship	Anterior Crossbite	Deep Overbite	Anterior Open Bite	Crossbite ^Ψ^	Posterior Crossbite	Scissor Bite	Midline Deviation	Crowding
***Asia (N = 13)***														
Chen et al. [25]	2015	Beijing, China	734				8.45%		0.95%		2.32%			
Zhou et al. [26]	2016	Xi’an, China	2235	66.31%	34.99% ^c^	2.46%	6.80%	37.58% ^β^	6.98%		7.56%		25.32%	
Zhou et al. [27]	2017	Shanghai, China	2335	83.90%	33.90% ^c^	2.30%	8.00%	63.70%	0.40%		0.30%	0.30%	26.60%	6.50%
Zhang et al. [28]	2017	Hong Kong, China	495		38% ^f^		12%		1%		1%			
Duraisamy V et al. [29]	2014	South India	187	63.60%	20.90%				15.00%	10.70%				17.10%
Khan R et al. [30]	2014	East Lucknow Region, India	453		9.10% ^c^			22.10% ^β^	0.70%	1.80%			2.60%	18.30%
Lochib et al. [31]	2015	Faridabad City, Haryana, India	1000				0.10%				0.80%			0.30%
Fernandes et al. [32]	2017	Mehsana District, North Gujarat, India	383		5.20% ^c^			15.90% ^β^	2.90%		1.60%			1.30%
Elene et al. [33]	2021	Tbilisi, Georgia	396	49.80%				10.70%	6.90%	7.00%				
Sasaki et al. [34]	2022	Japan	477	53.50%	19.90%		7.70%	19.90%	7.80%		0.00%	0.00%		10.90%
Lin et al. [13]	2023	Huizhou, China	1454	68.30%	12.5% ^d^	2.70%	7.80%	48.60%	1.20%		0.10%	0.10%	8.00%	10.50%
Otsugu et al. [35]	2023	Osaka, Japan	503	62.00%	27.8% ^d^		9.50%	23.1% ^γ^	7.20%				40.40%	11.50%
Abdellatif et al. [36]	2024	Riyadh City, Saudi Arabia	709	59.10%	25.11% ^d^	2.96%	5.78%	26.23%	5.50%		8.00%		17.07%	14.10%
***Europe (N = 4)***														
Berneburg M et al. [21]	2010	Southwest Ger- many	2016	61.50%	16.50% ^b^	3.30%	1.30%	25.50% ^α^	4.60%		10.70%			
Sepp H et al. [37]	2019	Estonia	390		12.10% ^d^		2.30%	27.40% ^γ^	3.10%		17.40%	0.50%		
Davidopoulou et al. [38]	2022	Greece	1222		37.80%		4.80%	40.10%			10.00%			
Kongo et al. [39]	2023	Shkodër, Albania	389		40.4% ^a^	7%	9.30%	39.3% ^α^	5.40%		23.40%		29%	
***South America (N = 27)***														
Bauman JM et al. [20]	2018	Brazil	6855	63.20%	22.80%	7.00%	3.10%	10.80%	11.10%		18.70%			
Normando TS et al. [40]	2015	Belém, Pará, Brazil	652	81.44%	13.30%		4.60%	23.20%	7.50%		6.00%			
Sousa et al. [41]	2013	Camp- ina Grande, Brazil	732	62.40%	42.60% ^a^		2.20%	19.30% ^α^	21.00%		11.60%			
Gomes MC et al. [42]	2014	Camp- ina Grande, Brazil	843	64.80%	43.40% ^a^		2.70%	18.70% ^α^	21.00%		12.10%			
Machado et al. [43]	2020	Teres- ina, Piauí, Brazil	566	51.20%	15.20% ^a^	4.40%	3.50%	12.70%	5.50%		7.10%			
Assis et al. [44]	2020	Aiqua- ra, Bahia, Brazil	148	69.59%	34.50%	17.60%	6.80%	8.10%			20.90%			
Carvalho et al. [45]	2013	Belo Horizonte, Minas Gerais, Brazil	1069	46.20%	10.50% ^a^		6.70%	19.70% ^α^	7.90%		13.10%			
Corrêa-Faria et al. [46]	2013	Diamantina, Minas Gerais, Brazil	381	32.50%			10.00%		12.30%		10.00%			11.50%
Ramos-Jorge J et al. [47]	2015	Diamantina, Minas Gerais, Brazil	451	28.40%	8.40% ^c^		0.90%		9.50%		20.40%			
Márcio et al. [48]	2021	Diamantina, Minas Gerais, Brazil	381	43.00%	15.70% ^c^		17.30%		10.50%		8.90%			
Jabbar et al. [49]	2011	São Paulo City, Brazil	911		39.50% ^a^		1.80%							
Bueno SB et al. [50]	2013	Campo Limpo Paulista, Brazil	138		15.20% ^e^			18.10%	20.30%		15.90%			
Abanto J et al. [51]	2015	Diadema, São Paulo, Brazil	1215	37.40%	7.50% ^c^		6.10%		22.10%		1.80%			
Diego et al. [52]	2021	Araras, São Paulo, Brazil	571		31.50% ^a^									
Antunes LA et al. [53]	2015	Friburgo, State of Rio de Janeiro, Brazil	606	48.60%	14.00% ^c^		2.80%	6.40% ^β^	34.80%					
Fernanda et al. [54]	2021	Florianopolis, Brazil	1050	36.70%	67.20% ^c^				11.40%		21.40%			
Jéssica et al. [55]	2021	Florianópolis, Brazil	570		30.00% ^c^				21.40%					
Motta-Rego et al. [56]	2022	Diamantina, Brazil	347		41.50%									
Souto-Souza et al. [57]	2022	Diamantina Minas Gerais, Brazil	123	60.16%										
Silva et al. [58]	2022	Teresina, Brazil	834	56.8%										
Scarpelli et al. [59]	2013	Belo Horizonte, Brazil	1632	46.70%										
Clementino et al. [60]	2015	Campina Grande, Brazil	843	63.20%										
Corrêa-Faria et al. [61]	2018	Brazil	5278	63.30%										
Perazzo et al. [62]	2020	Paraiba, Brazil	769	57.70%					15.20%					
Souto-Souza et al. [63]	2020	Diamantina, Brazil	384	58.30%										
Torres et al. [54]	2021	Florianopolis, Brazil	1050	36.70%	67.2% ^c^				11.40%		21.40%			
Huamán Mendoza et al. [64]	2023	Huancavelica, Peru	120	50.80%	0.8% ^c^	15.00%	2.50%	27.50%	6.70%					
***Africa (N = 1)***														
Mtaya M et al. [65]	2017	Dar es Salaam City, Tanzania	253	32.50%	1.20% ^e^		5.50%	6.30% ^β^	18.60%		1.20%		7.90%	0.80%

Diagnosis for increased overjet: ^a^ >2 mm, ^b^ >2.5 mm, ^c^ >3 mm, ^d^ >4 mm, ^e^ >5 mm, ^f^ >6 mm; diagnosis for deep overbite: ^α^ >2 mm, ^β^ >3 mm, ^γ^ >4 mm. ^Ψ^ crossbite: include anterior and posterior crossbite.

**Table 2:** Summary of terminal plane relationship of the second primary molars and canine relationship in the included studies.

Region, Authors	Year	Study Site	Total Sample Size	Terminal Plane Relationship of the Second Primary Molars				Canine Relationship		
				Flush Terminal Plane	Distal Step	Mesial Step	Bilateral Symmetry	Class I	Class II	Class III
***Asia (N = 9)***										
Zhou et al. [27]	2017	Shanghai, China	2335	38.70%	11.30%	38.50%	88.40%	57.00%	32.40%	9.70%
Zhang et al. [28]	2017	Hong Kong, China	495	Left-64% Right-65%	Left-9% Right-9%	Left-27% Right-26%		Left-79% Right-75%	Left-5% Right-6%	Left-16% Right-19%
Khan R et al. [30]	2014	East Lucknow Region, India	453	62.30%	6.40%	31.30%		91.60%	8.40%	0.00%
Lochib et al. [31]	2015	Faridabad City, Haryana, India	1000	65.10%	2.40%	12.80%	81.20%			
Fernandes et al. [32]	2017	Mehsana District, North Gujarat, India	383	55.40%	1.30%	43.30%		95.80%	2.10%	2.10%
Elene et al. [33]	2021	Tbilisi, Georgia	396					52.70%	21.20%	1.60%
Elene et al. [66]	2022	Tbilisi, Georgia	396	52.70%	21.20%	26.10%				
Lin et al. [13]	2023	Huizhou, China	1454	58.30%	16.70%	25%		63.80%	23.70%	12.50%
Abdellatif et al. [36]	2024	Riyadh City, Saudi Arabia	709	42.03%	2.96%	55.01%		83.22%	9.59%	7.19%
***Europe (N = 4)***										
Berneburg M et al. [21]	2010	Southwest Germany	2016					72.60%	22.60%	4.80%
Sepp H et al. [37]	2019	Estonia	390	42.80%	33.60%	47.90%	75.10%	69.70%	5.60%	3.80%
Da-vidopoulou et al. [38]	2022	Greece	1222	Right-51.40% Left-54.40%	Right-24.40% Left-22.00%			Right-60.00% Left-63.20%	Right-35.20% Left-32.5%	Right-4.00% Left-3.50%
Kongo et al. [39]	2023	Shkodër, Northern Albania	389	Right-52.20% Left-50.10%	Right-29.60% Left-32.40%	Right-17.70% Left-17.20%		Right-64.50% Left-62.20%	Right-22.60% Left-27.0%	Right-12.90% Left-10.80%
***South America (N = 5)***										
Bauman JM et al. [20]	2018	Brazil	6855					77.10%	16.30%	6.60%
Normando TS et al. [40]	2015	Belém, Pará, Brazil	652	9.40%	67.50%	4.50%				
Machado et al. [43]	2020	Teresina, Piauí, Brazil	566					Left-74.7% Right-74.0%	Left-16.8% Right-17.0%	Left-8.5% Right-9.0%
Assis et al. [44]	2020	Aiquara, Bahia, Brazil	148					66.20%	25.70%	8.10%
Alarcón-Calle et al. [67]	2022	Peru	160			83.60%				
***Africa (N = 1)***										
Mtaya M et al. [65]	2017	Dar es Salaam City, Tanzania	253	90.90%	0.80%	8.30%				

**Table 3:** Prevalence of malocclusion in different continents.

Variable		Asia		Europe		America		Africa		Worldwide		*p*-Value
		Mean (%)	SD	Mean (%)	SD	Mean (%)	SD	Mean (%)	SD	Mean (%)	SD	
	Malocclusion	61.81	10.72	61.50	#	52.69	13.37	32.50	#	54.83	13.41	0.144
Sagittal	Increased overjet	22.74	11.30	26.70	14.47	27.41	19.23	1.20	#	25.18	16.67	0.416
	Edge-to-edge incisor relationship	2.61	0.29	3.77	3.03	11.00	6.30			5.97	5.47	0.049 *
	Anterior crossbite	7.35	3.23	4.43	3.57	5.07	4.30	5.50	#	5.73	3.85	0.226
Vertical	Deep overbite	29.76	17.09	33.08	7.70	16.45	6.76	6.30	#	23.79	13.89	0.018 *
	Anterior open bite	4.71	4.37	4.46	0.97	14.68	7.69	18.60	#	10.07	7.91	0.000 *
Transversal	Crossbite ^Ψ^	6.50	4.47							6.50	4.47	
	Posterior crossbite	2.71	3.21	15.38	6.30	13.52	6.40	1.20	#	10.14	7.64	0.002 *
	Scissor bite	0.20	0.14	0.50	#					0.30	0.20	0.221
	Midline deviation	20.00	13.74	29.00	#			7.90	#	19.61	12.93	0.353
Spacing discrepancies	Crowding	10.06	6.34			11.50	#	0.80	#	9.35	6.35	0.421
Terminal plane relationship of the second primary molars	Flush terminal plane	54.40	10.66	48.95	5.40	9.40	#	90.90	#	52.33	19.39	0.164
	Distal step	7.80	6.96	29.27	5.41	67.50	#	0.80	#	17.56	19.55	0.034 *
	Mesial step	33.35	13.67	29.38	16.26	44.13	56.04	8.30	#	32.16	21.76	0.570
Canine relationship	Class I	76.22	17.84	66.83	5.18	72.57	5.68			72.49	12.74	0.463
	Class II	13.20	11.41	21.71	11.79	19.63	5.26			17.30	10.48	0.349
	Class III	6.35	6.59	6.05	3.90	7.83	1.12			6.60	4.76	0.690

* *p*  <  0.05. ^Ψ^ crossbite: include anterior and posterior crossbite. # No SD value is available due to the fact that the prevalence mean score is derived by only one study.

**Table 4:** Quality assessment of the included studies with the modified Newcastle–Ottawa Scale.

Region, Authors	Year	Study Site	Item						Total	Quality
			1	2	3	4	5	6		
***Asia (N = 14)***										
Chen et al. [25]	2015	Beijing, China	0	1	1	2	2	1	7	Good
Zhou et al. [26]	2016	Xi’an, China	1	1	0	2	2	1	7	Good
Zhou et al. [27]	2017	Shanghai, China	1	1	0	2	2	0	6	Good
Zhang et al. [28]	2017	Hong Kong, China	1	1	1	2	2	1	8	Good
Duraisamy V et al. [29]	2014	South India	0	1	0	0	0	1	2	Poor
Khan R et al. [30]	2014	East Lucknow Region, India	1	1	0	2	0	1	5	Moderate
Lochib et al. [31]	2015	Faridabad City, Haryana, India	0	1	0	2	0	1	4	Moderate
Fernandes et al. [32]	2017	Mehsana District, North Gujarat, India	1	1	0	2	0	1	5	Moderate
Elene et al. [33]	2021	Tbilisi, Georgia	1	1	1	1	2	1	7	Good
Elene et al. [66]	2022	Tbilisi, Georgia	1	1	0	2	0	1	5	Moderate
Sasaki et al. [34]	2022	Japan	0	1	1	2	0	1	5	Moderate
Otsugu et al. [35]	2023	Osaka, Japan	0	1	1	2	0	1	5	Moderate
Lin et al. [13]	2023	Huizhou, China	1	1	1	2	2	1	8	Good
Abdellatif et al. [36]	2024	Riyadh City, Saudi Arabia	1	1	0	2	2	1	7	Good
***Europe (N = 4)***										
Berneburg M et al. [21]	2010	Southwest Germany	1	1	0	2	2	1	7	Good
Sepp H et al. [37]	2019	Estonia	1	1	0	2	0	1	5	Moderate
Davidopoulou et al. [38]	2022	Greece	1	1	1	2	2	1	8	Good
Kongo et al. [39]	2023	Shkodër, Northern Albania	1	1	0	2	2	1	7	Good
***South America (N = 28)***										
Bauman JM et al. [20]	2018	Brazil	1	1	1	2	2	1	8	Good
Normando TS et al. [40]	2015	Belém, Pará, Brazil	1	1	1	1	2	1	7	Good
Sousa et al. [41]	2013	Campina Grande, Brazil	0	1	0	2	0	1	4	Moderate
Gomes MC et al. [42]	2014	Campina Grande, Brazil	1	1	1	2	2	1	8	Good
Machado et al. [43]	2020	Teresina, Piauí, Brazil	0	1	1	2	0	1	5	Moderate
Assis et al. [44]	2020	Aiquara, Bahia, Brazil	1	1	0	2	0	1	5	Moderate
Carvalho et al. [45]	2013	Belo Horizonte, Minas Gerais, Brazil	1	1	1	1	2	1	7	Good
Corrêa-Faria et al. [46]	2013	Diamantina, Minas Gerais, Brazil	0	1	0	1	2	1	5	Moderate
Ramos-Jorge J et al. [47]	2015	Diamantina, Minas Gerais, Brazil	0	1	1	1	2	1	6	Good
Márcio et al. [48]	2021	Diamantina, Minas Gerais, Brazil	1	1	1	2	2	1	8	Good
Jabbar et al. [49]	2011	São Paulo City, Brazil	1	1	0	2	2	1	7	Good
Bueno SB et al. [50]	2013	Campo Limpo Paulista, Brazil	1	1	1	2	2	1	8	Good
Abanto J et al. [51]	2015	Diadema, São Paulo, Brazil	0	1	0	2	2	1	6	Good
Diego et al. [52]	2021	Araras, São Paulo, Brazil	1	1	0	2	2	1	7	Good
Antunes LA et al. [53]	2015	Friburgo, State of Rio de Janeiro, Brazil	1	1	1	2	2	1	8	Good
Fernanda et al. [54]	2021	Florianopolis, Brazil	1	1	1	2	2	1	8	Good
Jéssica et al. [55]	2021	Florianópolis, Brazil	1	1	1	2	2	1	8	Good
Alarcón-Calle et al. [67]	2022	Peru	1	1	0	2	2	1	7	Good
Souto-Souza et al. [57]	2022	Diamantina Minas Gerais, Brazil	1	1	1	2	2	1	8	Good
Silva et al. [58]	2022	Teresina, Brazil	1	1	1	2	2	1	8	Good
Motta-Rego et al. [56]	2022	Diamantina, Brazil	1	1	1	2	2	1	8	Good
Scarpelli et al. [59]	2013	Belo Horizonte, Brazil	1	1	1	2	2	1	8	Good
Clementino et al. [60]	2015	Campina Grande, Brazil	1	1	1	2	2	1	8	Good
Corrêa-Faria et al. [61]	2018	Brazil	0	1	0	2	2	1	6	Good
Perazzo et al. [62]	2020	Paraiba, Brazil	1	1	0	2	2	1	7	Good
Souto-Souza et al. [63]	2020	Diamantina, Brazil	0	1	1	2	2	1	7	Good
Torres et al. [54]	2021	Florianopolis, Brazil	1	1	1	2	0	1	6	Good
Huamán Mendoza et al. [64]	2023	Huancavelica, Peru	0	1	0	2	0	1	4	Moderate
***Africa (N = 1)***										
Mtaya M et al. [65]	2017	Dar es Salaam City, Tanzania	0	1	1	2	0	1	5	Moderate

Item 1, representativeness; item 2, sample size; item 3, non-respondents; item 4, ascertainment of risk factor (diagnosis); item 5, outcome assessment; item 6, statistics.

## Data Availability

The datasets generated during and/or analyzed during the current study are available from the corresponding author on reasonable request.

## Supplementary Materials

The following supporting information can be downloaded at: https://www.mdpi.com/article/10.3390/healthcare12131321/s1, Supplementary File S1: PRISMA checklist [91]; Supplementary File S2: Newcastle–Ottawa Scale adapted for cross-sectional studies.
