# Supplementary material for: Prevalence of Malocclusion Traits in Primary Dentition, 2010–2024: A Systematic Review
Source: Healthcare (Basel). 2024 Jul 2;12(13):1321. doi: 10.3390/healthcare12131321 (PMC11241413; doi:10.3390/healthcare12131321)
Supplement: Supplementary file 1 [file healthcare-12-01321-s001.zip › Supplementary File S2. NOS.pdf]

Supplementary File S2

NEWCASTLE - OTTAWA QUALITY ASSESSMENT SCALE

(adapted for cross sectional studies)

Selection: (Maximum 5 scores)

1) Representativeness of the cases:

a) Truly representative of the HCC patients (consecutive or random sampling of cases). 1 score

b) Somewhat representative of the average in the HCC patients (non-random sampling) . 1 score

c) Selected demographic group of users. 0 score

d) No description of the sampling strategy. 0 score

2) Sample size:

a) Justified and satisfactory (≥ 400 HCC included). 1 score

b) Not justified (<400 HCC patients included). 0 score

3) Non-Response rate

a) The response rate is satisfactory (≥95%). 1 Score

b) The response rate is unsatisfactory (<95%), or no description. 0 Score

4) Ascertainment of the screening/surveillance tool:

a) Validated screening/surveillance tool. 2 scores

b) Non-validated screening/surveillance tool, but the tool is available or described. 1 score

c) No description of the measurement tool. 0 score

Comparability: (Maximum 1 stars)

1) The potential confounders were investigated by subgroup analysis or multivariable analysis.

a) The study investigates potential confounders. 1 score

b) The study does not investigate potential confounders. 0 score

Outcome: (Maximum 3 stars)

1) Assessment of the outcome:

a) Independent blind assessment. 2 scores

b) Record linkage. 2 scores

c) Self report. 1 score

d) No description. 0 score

2) Statistical test:

a) The statistical test used to analyze the data is clearly described and appropriate. 1 score

b) The statistical test is not appropriate, not described or incomplete. 0 score
